# Supplementary material for: Biophysical Modeling of SARS-CoV-2 Assembly: Genome Condensation and Budding
Source: Viruses. 2022 Sep 20;14(10):2089. doi: 10.3390/v14102089 (PMC9611094; doi:10.3390/v14102089)
Supplement: Supplementary file 1 [file viruses-14-02089-s001.zip › SI.pdf]

Supplementary Materials: Biophysical Modeling of SARS-CoV-2 Assembly: Genome Condensation and Budding

Siyu Li [1,*] and Roya Zandi [2,*]

1. Supplementary Figure

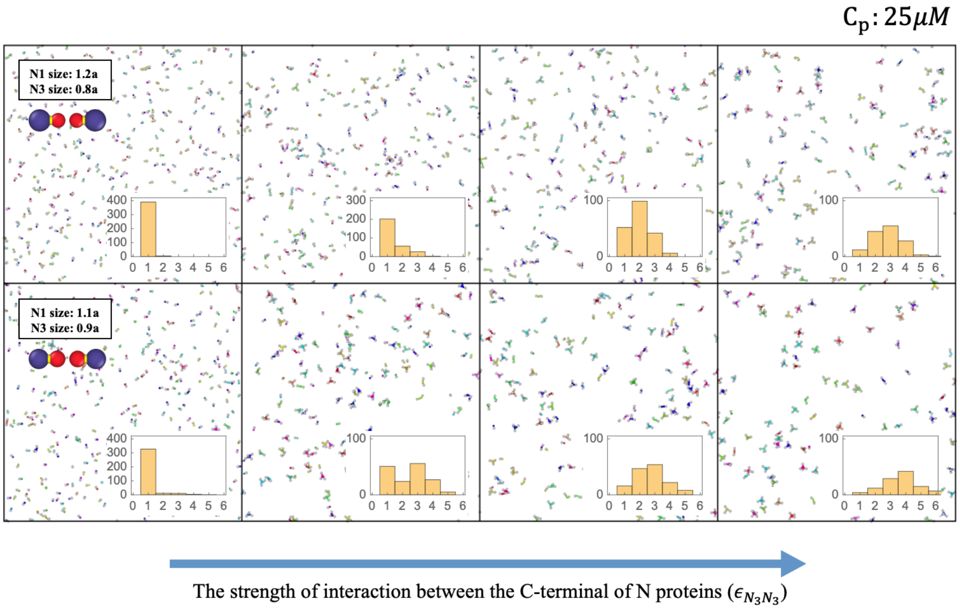

Figure S1. Dimerization of the N proteins for various domain sizes and the strengths of interaction between the C-terminals. The upper row shows N proteins with an N-terminal size of 1.2a and a C-terminal size of 0.8a, where a is the system length corresponding to 3 nm. The size of N-terminal and C-terminal are 1.1a and 0.9a, respectively, in the lower row. The size of the linker region N2 is kept constant and is 0.5a. The N-terminals of N proteins are colored gray and the C-terminals of N proteins have different bright colors based on their cluster index (see SI Fig. S2 for the definition of the cluster index). The histograms in the figure show the count of the N protein oligomers. For the weak C-terminal interactions, we see that nearly all N proteins are monomers. As the interaction strength increases, more dimers and trimers form in solution. The interaction strengths $\epsilon_{N_3N_3}$ are 10, 15, 20 and 50 from left to right. We find that dimers are the dominant structure at $\epsilon_{N_3N_3} = 20$.

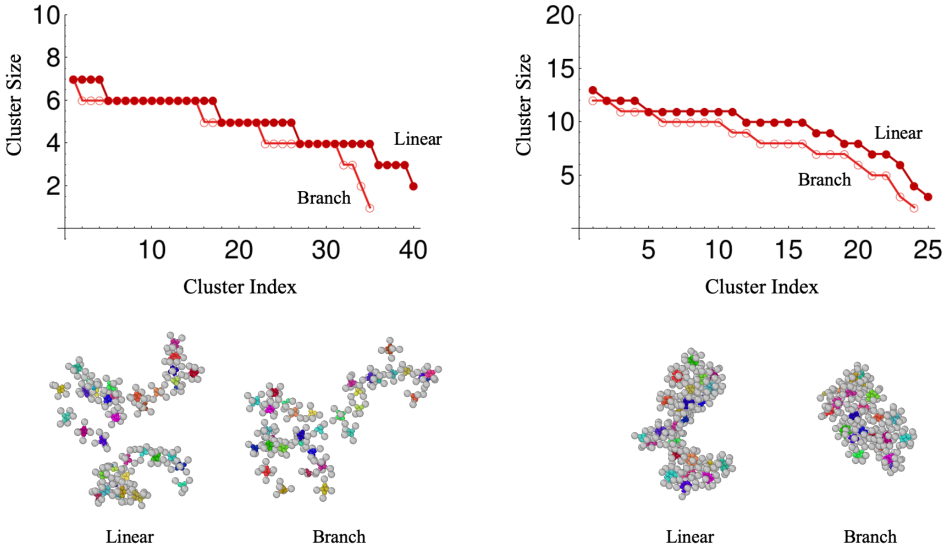

Figure S2. Cluster analysis of NPCs along RNP for weak (left) and strong (right) RNA-N protein interactions. We recall that when the distance between the N-terminals of the N proteins are less than a cut-off distance, they are defined as a cluster assigned with a specific color and a unique cluster index. The plots display the number of N proteins in each cluster, where the clusters are sorted by their sizes. RNA is wrapped around the N proteins and is not shown in the figure.

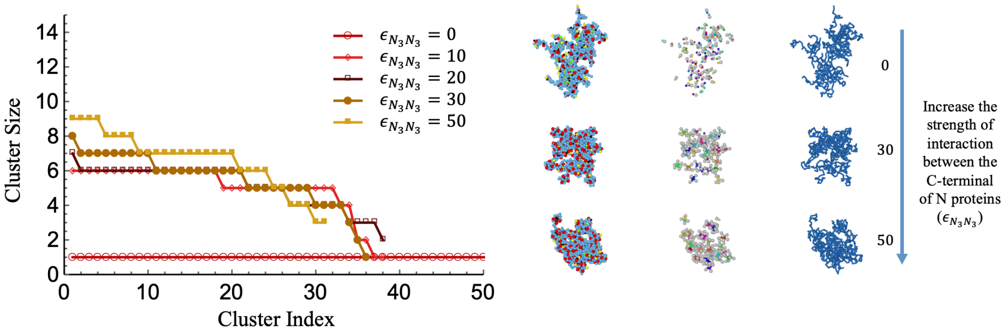

Figure S3. RNP condensation for various strengths of interactions between N proteins. The left figure shows the cluster analysis for various N3-N3 interactions while the right figures display the structures of RNP, NPCs and RNA from left to right. The strength of interaction between the C-terminal of N proteins ($\epsilon_{N_3N_3}$) increases from top to bottom. As the strength of the N-N interaction increases, the number of N proteins in each cluster increases, but the changes are small. Also, the size of RNP remains more or less the same. We note again that the N proteins in each cluster have the same color and a unique cluster index, and that the clusters are sorted by their sizes. The other parameters used are $Z_g = -2$, and N protein concentration is 25μM.

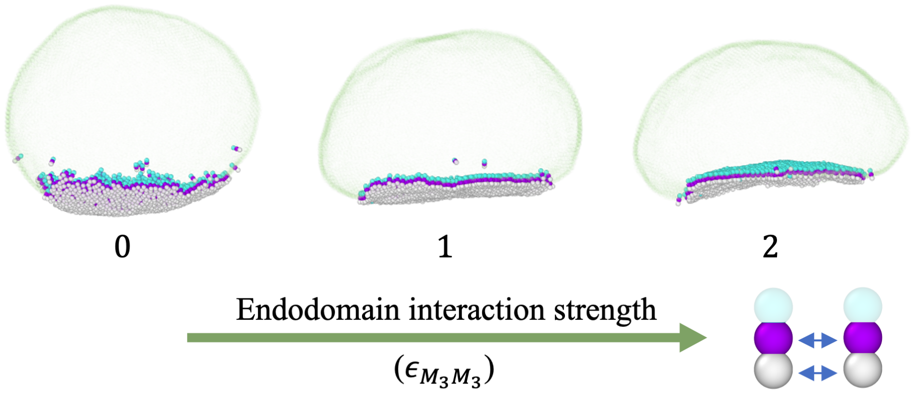

Figure S4. Illustration of the role of the spontaneous curvature in the budding process. The M protein has a cylindrical structure (no spontaneous curvature). All three spherical beads making the M protein have the same diameter. In particular, the transmembrane particle (purple) has the same size as endodomain particle (white). The transmembrane interaction strength is $\epsilon_{M_2M_2} = 1$ and the endodomain (white) interaction strength $\epsilon_{M_3M_3}$ is 0, 1 and 2 from top to bottom. There is no budding in the absence of spontaneous curvature.

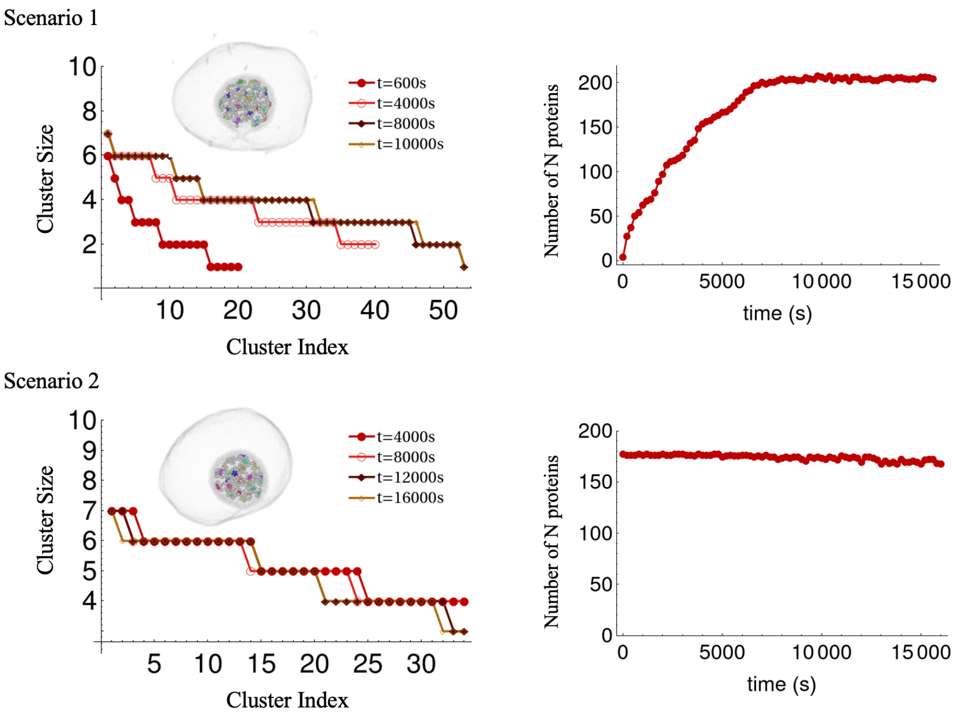

Figure S5. Cluster analysis of the N proteins along RNP during the budding. The left figures show the number of N proteins in different clusters along RNP during the budding process vs. the cluster index for scenario 1 (top) and scenario 2 (bottom). The insets show the configurations for which the clusters along RNPs were studied. Recall that the N proteins in the same cluster have the same color and index number, and that they are sorted by the size of the clusters. The right figures show the total number of N proteins attached to RNA as a function of time during the budding process. Since in the scenario 1, RNP condensation and budding take place at the same time, the number of N proteins increases as a function of time. However, in scenario 2, RNP has already formed and then has been transported near ERGIC before budding.

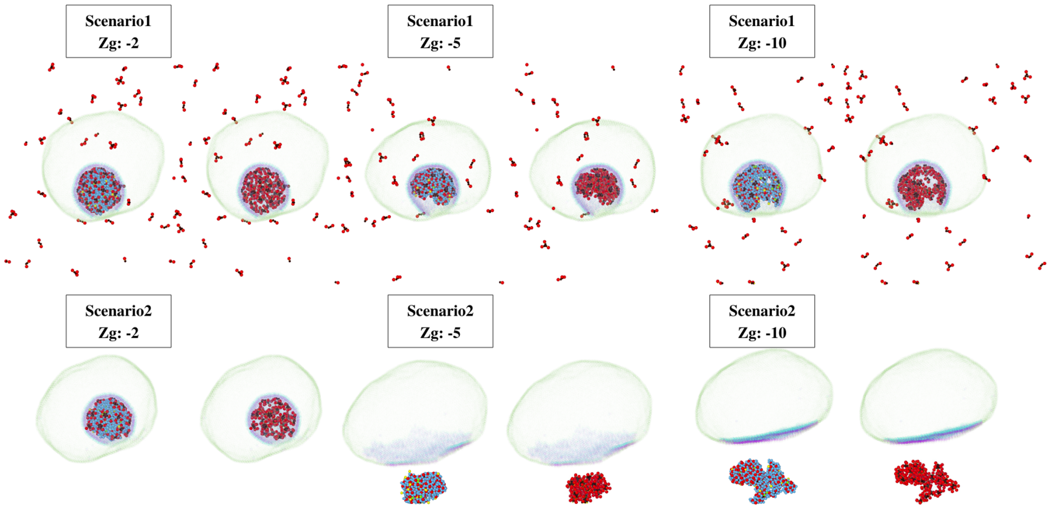

Figure S6. RNP budding with various N protein-RNA interactions for scenarios 1 (top) and 2 (bottom) at $t = 16,000s$**.** There are three pairs of figures in each row. The left figure in each pair shows RNP and the right one shows the N proteins only. In scenario 1, ERGIC is initially located in the vicinity of RNA and the N proteins, and the RNA-N protein condensation and budding happen at the same time. In scenario 2, pre-condensed RNA-N proteins are placed near ERGIC, which later bud into a vesicle. See the assembly and budding movies SI Video S6 and Video S7. The figure also shows the simulations at various genome effective charge densities with $Z_g = -2, -5, -10$ from left to right. We find that when the genome effective charge density is small, both scenarios (1 and 2) are plausible resulting in the formation of viral particles. However, as the genome effective charge density increases, more N proteins get absorbed to RNA, which makes it difficult for them to reorient and expose their C-terminal domains to interact with M proteins.

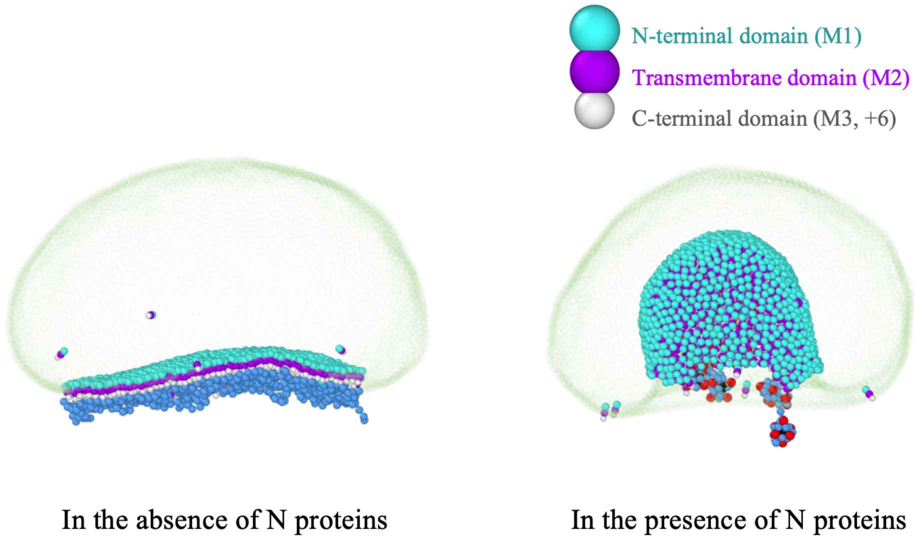

Figure S7. Snapshots of the simulations of the RNP budding when the endodomains of M proteins are positively charged. The figure illustrates that the attractive electrostatic interactions between RNA and the M proteins is not sufficient for the RNA budding (left figure), and the presence of N proteins are essential for RNA packaging and budding (right figure). Note that a stronger N3-M3 interaction ($\epsilon_{N_3M_3} = 20$) is required to trigger the budding compared to the neutral M protein case where a much weaker $\epsilon_{N_3M_3} = 10$ results in the budding and assembly of the virus (Fig. 7). Both simulations are performed for 8000s and other parameters used are $L = 800$, $\epsilon_{McMc} = 1$, $\epsilon_{M_2M_2} = 1$, $\epsilon_{M_3M_3} = 0$, $Z_g = -2$ and $\epsilon_{N_3N_3} = 20$. We recall that the electrostatic repulsion between M3 and M3 is assumed to be screened and thus is zero. Note that for shorter chains, it's possible to package RNA in the absence of N proteins, but not relevant to the case of coronaviruses.

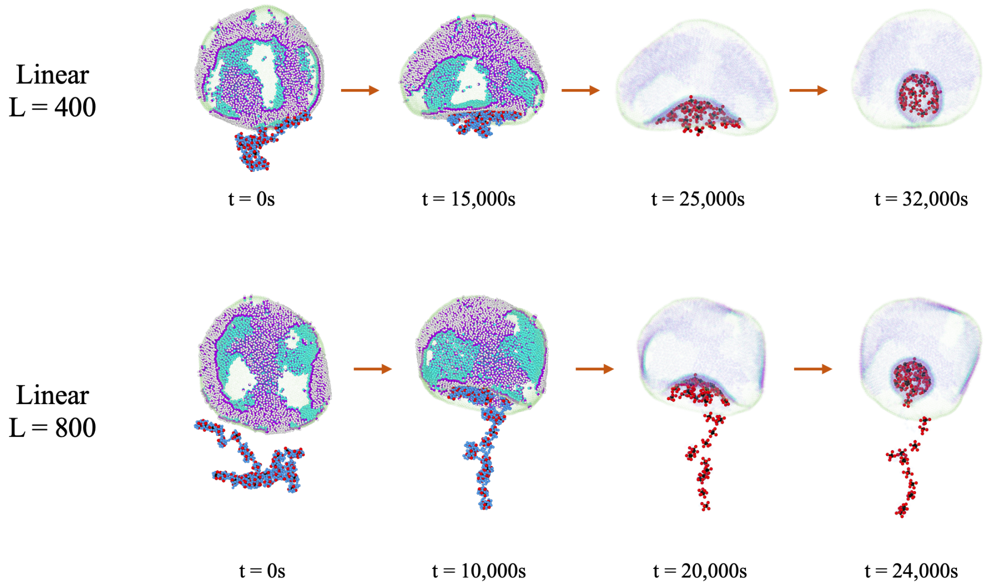

Figure S8. Snapshots of simulations of the RNP budding using a linear RNA. Note, only shorter chains with $L = 400$ (upper row) can be completely encapsidated. The M proteins are initially randomly distributed, and ERGIC has been fully relaxed. The 25µM protein concentration was used to condense RNA. The resulting RNP is then located near ERGIC. As illustrated in the figure, when $L = 800$ (the bottom row), the chain cannot be fully packaged and a part of the chain will remain outside of the envelope. For the last two figures in each row, we show only the configuration of the N proteins. RNA has wrapped around the N proteins but is not shown in the figure. Note that RNP could be packaged if RNA were modeled as a branched polymer with the same length ($L = 800$). The other parameters used are $\epsilon_{McMc} = 1$, $\epsilon_{M_2M_2} = 1$, $\epsilon_{M_3M_3} = 0$, $Z_g = -2$, $\epsilon_{N_3N_3} = 20$, and $\epsilon_{N_3M_3} = 10$.

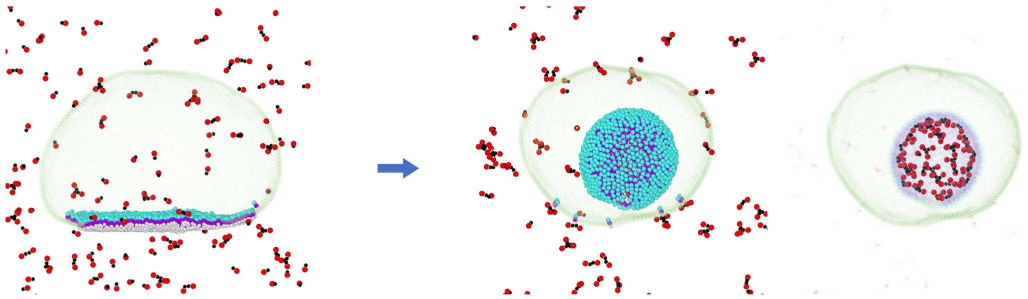

Figure S9. Formation of the virus like particles through the budding of N proteins in the absence of RNA. The parameters used are $\epsilon_{M_2M_2} = 1$, $\epsilon_{M_3M_3} = 0$, $\epsilon_{N_3N_3} = 20$, and $\epsilon_{N_3M_3} = 10$. Note that in the absence of the N proteins, the M proteins cannot bud with the same M-M interaction strength parameters.

Supplementary Video

Video S1. RNA condensation by the N proteins

The N proteins of SARS-CoV-2 condense RNA. The concentration of proteins is 25μM. RNA is modeled as a branched polymer with the number of branch points N = 30 and the genome effective charge density $Z_g = -5$.

Video S2. The impact of the secondary structure of RNA on its condensation

N proteins condense RNA with different number of branch points. The movies show RNP with the genome in blue and N proteins in red and black. The branch points are in green and the end points are in yellow. The total running time is 5000s. The number of branch points from left to right is N = 0, 10, 20, and 30.

Video S3. N proteins condense a linear genome for various N protein concentrations and effective genome charge densities

N proteins condense RNA modeled as a linear polymer for different protein concentrations and effective genome charge densities. The movies show RNP with genome in blue and N proteins in red and black. The total running time is 5000s. The genome effective charge density increases from left to right with $Z_g = -2$, -5, and -10.

Video S4. N proteins condense RNA at various N protein concentrations and effective genome charge densities

N proteins condense RNA modeled as a branched polymer at various protein concentrations and effective genome charge densities. The movies show RNP with RNA in blue and N proteins in red and black. The number of branch points is N = 30. The branched points are colored green and the end points are colored yellow. The total running time is 5000s. The genome effective charge density increases from left to right with $Z_g = -2$, -5, and -10.

Video S5. The role of the M protein interactions in budding at the ERGIC membrane

Budding through the ERGIC membrane for various interaction strengths between the M protein transmembrane domains on the one hand and between the endodomains on the other hand. The lipid-lipid interaction strength is kept constant $\epsilon_{McMc} = 1$ for all simulations. The membrane stretching modulus is $k_s = 20a^{-2}$ and the bending modulus is $k_b = 20$. The total running time is 3000s.

Video S6. RNP budding through the ERGIC membrane for scenario 1

RNP budding through the ERGIC membrane for scenario 1. The N proteins interact with the RNA and form many clusters while simultaneously interacting with the M proteins embedded in the lipid membrane. Thus, the RNP condensation and budding occur simultaneously at the ERGIC membrane.

Video S7. RNP budding through the ERGIC membrane for scenario 2

RNP budding through the ERGIC membrane for scenario 2. First, the N proteins interact with the RNA to form the condensed RNP complex, which later interacts with the M proteins that are embedded in the lipid membrane. Budding through the intercellular membrane then completes virus formation.

Video S8. RNP budding through the ERGIC membrane for the randomly distributed M proteins

RNP budding through the ERGIC membrane movie for the randomly distributed M proteins. See SI Video S7 for the budding through a circular cap made of M proteins.
